# The Model Man

**Published:** 1857-11

**Authors:** 


### "THE MODEL MAN."

An individual writes to *Life Illustrated* as follows:

"I drink no kind of liquid, except water. I use no kind of medicine, tobacco, or snuff. I eat no kind of meat, fish, fowl, salt, pepper, vinegar, mustard, spices, or pickles. I belong to no party or sect. I never swear, bet, play at cards, or gamble in any way. I never go to law, fight, or interfere with my neighbors' private affairs. I never visit any theatre, drinking saloon, gambling-house, brothel, or useless shows. I never read any novels, or follow any fashion or opinion, unless I think it is perfectly right. I claim the right to investigate any subject which presents itself, and then to judge for myself. I also allow the same privilege to others. I violate no moral or physical law, that I know of. I despise no one for his poverty, nor do homage to any one for his wealth. Whenever the people can thus reform themselves, we then can get rid of the present expensive and corrupt governments, whereby it takes one-half of the people to govern and regulate the other half of society. We should then want no law, soldiers, police jails, or governments, for every one would be a law of him self."

We will venture to say that the author of the foregoing is a self-righteous, self-conceited ignoramus, without influence where he resides, without a Bible, without religion, and "without hope in the world." If all men were made after his fashion, no human government would last an hour; for his rule of action is what *he* thinks "*is perfectly right*," and his standard of justice would be the laws he would make "of himself." And what if he does not eat meat, fish, or fowl, he eats what he likes—just like any other pig. The conceit of a fool is as amazing as the cowardice of a braggadocio!

